# Fusion of Optimized Indicators from Advanced Driver Assistance Systems (ADAS) for Driver Drowsiness Detection

**DOI:** 10.3390/s140101106

**Published:** 2014-01-09

**Authors:** Iván G. Daza, Luis M. Bergasa, Sebastián Bronte, J. Javier Yebes, Javier Almazán, Roberto Arroyo

**Affiliations:** Department of Electronics, University of Alcalá, Alcalá de Henares, Madrid 28871, Spain; E-Mails: ivan.garciad@uah.es (I.G.D.); bergasa@depeca.uah.es (L.M.B.); sebastian.bronte@depeca.uah.es (S.B.); javier.yebes@depeca.uah.es (J.J.Y.); javier.almazan@depeca.uah.es (J.A.)

**Keywords:** ADAS, driver drowsiness, driver physical measures, driving performance measures, PERCLOS, data fusion, neural networks, binary classification, third generation simulator

## Abstract

This paper presents a non-intrusive approach for monitoring driver drowsiness using the fusion of several optimized indicators based on driver physical and driving performance measures, obtained from ADAS (Advanced Driver Assistant Systems) in simulated conditions. The paper is focused on real-time drowsiness detection technology rather than on long-term sleep/awake regulation prediction technology. We have developed our own vision system in order to obtain robust and optimized driver indicators able to be used in simulators and future real environments. These indicators are principally based on driver physical and driving performance skills. The fusion of several indicators, proposed in the literature, is evaluated using a neural network and a stochastic optimization method to obtain the best combination. We propose a new method for ground-truth generation based on a supervised Karolinska Sleepiness Scale (KSS). An extensive evaluation of indicators, derived from trials over a third generation simulator with several test subjects during different driving sessions, was performed. The main conclusions about the performance of single indicators and the best combinations of them are included, as well as the future works derived from this study.

## 1. Introduction

Of all the problems related to transport, safety is the one that has a greater impact on the everyday life of citizens. In addition, it significantly affects the majority of socio-economic indicators. A great deal of effort has been made in recent years in the EU on programs of action for road safety, reducing traffic crashes by 40% from 2001 to 2010 [1]. However, the situation is still far from satisfactory. In 2010, there were almost 31,000 deaths and 1,400,000 crashes on the roads of Europe of the twenty-seven states (27 EU), which represents an annual cost of approximately 200,000 million Euros, equivalent to 2% of the EU GNP (Gross Domestic Product) [2]. The current white paper on transport in the EU, published in 2011, aims to halve the number of road deaths by 2020 [3].

Recent studies have identified the inattention of the driver as the main cause of crashes. Additionally, the National Highway Traffic Safety Administration (NHTSA) estimates that approximately 25% of police-reported crashes involve some form of driver inattention [4]. One common definition of driver inattention is given in [5]: “Driver inattention represents diminished attention to activities that are critical for safe driving in the absence of a competing activity.” There are different categories for the driver attention state, but in practice, two main categories are proposed: (1) distraction and (2) fatigue. In this paper, we will focus on the fatigue category.

The term fatigue refers to a combination of symptoms, such as impaired performance and a subjective feeling of drowsiness. Even with the intensive research that has been performed so far, the term fatigue still does not have a universally accepted definition [6]. The European Transport Safety Council (ETSC) defines fatigue as tiredness concerning the inability or disinclination to continue an activity, generally because the activity has been going on for too long. Besides, it establishes drowsiness as one external representation of fatigue, being the most important for driving. For the purpose of this paper, the terms drowsiness and fatigue will be employed interchangeably, unless otherwise stated. Therefore, some studies carried out by ETSC conclude that there are four drowsiness levels, based on user behavior [7]: completely awake, moderately awake, drowsy and severely sleepy. In this paper, we will group them into two levels, alert and drowsy, in order to reduce complexity and to obtain generalized results by using binary classifiers. Fatigue during driving has been shown to result in a greatly increased risk of suffering a collision. In particular, [8] have shown that driving with fatigue raises the accident risk between four to six times, compared to alert driving. In fact, most of the research works carried out so far concluded that aspects related to fatigue were present in 15%–20% of the crashes [9]. As a consequence, the development of systems capable of monitoring drivers' fatigue state in real-time and capable of warning them just before they fall asleep is vital to prevent crashes.

In the last few decades, diverse techniques have been developed for monitoring driver drowsiness. According to the state-of-the-art, there are five main categories depending on the type of measures used in the detection: driver biological, subjective report, driving performance, driver physical and hybrid measures [10].

The first category directly measures the biological signals on the driver's body. The most important signals are: electroencephalography (EEG), electrocardiogram (ECG), electrooculography (EOG), surface electromyogram (sEMG), galvanic skin response (GSR) and respiration [11–13]. The main drawback of such signals is that conventional measuring instruments are intrusive. In addition, most of the biological patterns vary between individuals. However, some biological variables, such as ECG, GSR and respiration, can be measured with non-intrusive devices in the vehicle. These include sensors based on seat pressure [13], flying instrumented with electrodes [14] and nanosensors [15]. These devices are based upon the same technology as conventional devices, but the body contact is achieved indirectly by taking advantage of the driver's own position while driving. Their main limitation is that the quality of contacts is worse than those made clinically, which means using more sophisticated methods of signal analysis to remove noise and extract the relevant parameters. This causes a performance decrease that makes the proposal impossible in most cases. These signals are normally used in psychological studies and as the ground truth for other non-intrusive methods.

There are several subjective measurement scales in use. The two most common are the Sanford Sleepiness Scale (SSS), proposed by Hoddes *et al.* [16], and the Karolinska Sleepiness Scale (KSS), proposed by [17]. In the last few years, KSS has become the most employed tool for the subjective self-assessment of drowsiness. A supervisor asks the driver from time to time about his or her status while he or she is driving. The scale involves nine steps, of which every odd number is associated with a label as follows: KSS = 1 (extremely alert), 3 (alert), 5 (neither alert nor sleepy), 7 (sleepy, but not fighting sleep) and 9 (very sleepy, fighting sleep). KSS has been shown in several studies to suitably correlate with physiological signs of drowsiness [18,19]. However, KSS is recorded over relatively long time intervals, e.g., every 15 min, as a trade-off between high temporal resolution and avoiding intrusive feedback. As a consequence, KSS cannot record sudden drowsiness variations caused by different situations. In addition, this kind of approach requires that the driver frequently reports his or her state. Thus, both the drowsiness level result and the driver could cause interference. In brief, this is an intrusive measure that cannot be employed in the development of an automatic monitoring system to warn drivers before they fall asleep at the wheel in real time. As in the previous category, this measure is normally exploited for psychological studies of drowsiness and as the ground truth to test the performance of other proposals.

The measured signals on the vehicle reflect the actions of the driver, and therefore, by analyzing them, the driver's behavior can be indirectly characterized in a non-intrusive way. They are usually directly obtained from a simulator or from the internal sensors of a vehicle through the CAN (Controller Area Network) bus. The force on the pedals, changes in vehicle speed, steering wheel movements, the lateral position or lane changes are typically employed in this category [20,21]. Indicators of driver drowsiness may be formed by extracting relevant information from signals, such as those mentioned above. These indicators are scalar-valued functions that map given segments of signals onto numerical values. Several indicators, based on individual signals or a combination of them with different complexities have been exploited in the literature [22,23]. The advantage of this approach is that the signals have physical meaning, and their acquisition and posterior indicator computation is relatively easy. This is the reason why the few commercial systems currently available are mainly based on this category: Volvo's Driver Alert Control [24], Mercedes-Benz's Attention Assist [25], Lexus' Driver Monitoring System [26] or Saab's Driver Attention Warning System [27]. However, these systems usually require a training period for each person; thus, they are not applicable to occasional drivers. Additionally, they do not work in the detection of so-called micro-sleeps [28] (when a drowsy driver falls asleep for a few seconds on a straight road without changing the direction of the vehicle). On the other hand, from the authors knowledge, in the open literature, there are very few details available with regard to the mechanisms or parameters of commercial systems.

The physical measures approach is mainly based on monitoring the driver's face, employing cameras and image processing techniques to obtain some physical indicators: blink duration, blink rate, PERCLOS (Percentage of Eye Closure) [29], eye closure duration, nodding frequency, fixed gaze, *etc.* This approach is effective, because, on the one hand, driver drowsiness is exposed by the appearance of the driver's face and the activity of his head and eyes and, on the other hand, measures can be carried out in a non-intrusive way. However, they are normally used in research and simulated scenarios, but not in real ones, due to the problems of vision systems working in outdoor environments (lighting changes, sudden movements, *etc.*). Additionally, they do not work properly with users wearing glasses and need high computational requirements. In the state-of-the-art, different kinds of cameras and processing algorithms have been proposed: visible camera-based methods [13,30–32]; near-infrared (NIR) camera-based methods [33–35]; and methods based on stereo cameras [36–38]. Some of them are commercial products. These include the faceLAB [38], the DSS (Driver State Sensor) [38], the Smart Eye [37] and DFM (Driver Fatigue Monitoring) [31]. Numerous research groups have purchased commercial facial trackers for measuring physical signals related to the face and eyes, which allowed them to concentrate on exploring the drowsiness detection algorithms instead of the image acquisition and processing. However, these commercial supporters are closed source systems, and they only work properly under restricted conditions of the environment, significantly reducing their performance in real driving conditions. In addition, they usually require a hard calibration setup for each user. They lack of scientific publications in journals, and they do not provide the comprehensive tests and self-evaluations in rigorous scientific studies.

Regarding systems based on a single sensor, they usually have difficulties, due to the uncertainty of the observations associated with the measurements obtained in real conditions. The use of multi-sensor systems reduces the uncertainty and ambiguity of data obtained by a single source. Furthermore, the combination of indicators from different sensors, e.g., combining driver physical with driving performance measures, increases the confidence of drowsiness detection. These proposals are known as hybrid measurement systems. A review of the most representative works in this area shows that this strategy has been exploited in recent years [39]. Among them, we highlight the work of [22], where they presented some artificial neural networks (ANNs) to analyze vehicle parameter data (lane position, steering-wheel angle) and an eye-closure indicator computed from camera images (PERCLOS) in order to infer driver drowsiness without optimizing the indicators. It was reported that the system had an accuracy of 88%, with a false-alarm rate of 9% in simulation. In [40], a proposal called PERCLOS+ was presented, where PERCLOS was combined with lane deviation indicators. Based on simulation and preliminary on-road tests, their proposal was found to be more robust than only using PERCLOS. This work was focused on long-term drowsiness estimation technology, as opposed to our approach with real-time drowsiness detection technology. Another interesting work is the one presented in [23], where a variety of state-of-the-art optimized indicators based on driving signals are evaluated and combined using ANNs with a mathematical model of drowsiness. They showed that a nonlinear combination of indicators based on driving behavior with a model of drowsiness significantly improved driver fatigue detection, yielding a score of 0.83 in simulation. In the conclusion, they suggested that it might be worthwhile to include an indicator based on eye closure data.

This paper presents a non-intrusive approach for monitoring driver drowsiness employing the fusion of several optimized indicators based on driver physical and driving performance measures in simulation. This work is focused on real-time drowsiness detection technology rather than on long-term sleep/awake regulation prediction technology. The main goals of our proposal are: (1) to develop our own vision system in order to obtain robust and optimized driver physical indicators able to be used in simulators and future real vehicles; (2) to evaluate the fusion of several indicators using a neural network and a stochastic optimization method to obtain the best combination for the simulated scenarios; (3) to propose a new method for ground truth generation based on a supervised KSS; and (4) to validate our proposal by using a third generation simulator and different drivers.

The remainder of the paper is organized as follows. In Section 2, the dataset and ground truth methodology is described. Section 3 presents our drowsiness detection proposal. Section 4 explains the driver indicators and Section 5 the driving indicators employed in the proposed methodology. The optimization of indicators is studied in Section 6, and the data fusion process is analyzed in Section 7. Experimental results are drawn in Section 8. Finally, Section 9 concludes the paper and depicts some further work.

## 2. Dataset and Ground Truth

The dataset is composed of several sequences collected in a realistic driving simulator. The main goal of the simulated experiments was to infer drowsiness in the test subjects attending to different strategies, *i.e.*, sleep deprivation, day time and driving operation time and driver/driving behavior analysis in extreme conditions. A total of nine drivers participated in the experiments. They were recruited from the Spanish national register of professional drivers. They had to be frequent drivers, driving at least 5,000 km a year, not wearing glasses and not suffering from habitual sleep disturbances. Before participation, the subjects were informed and signed a consent form. Conditions in simulated roads for the different driving trials are shown in Table 1.

### 2.1. Trials in the Simulated Scenario

**Figure 1.**
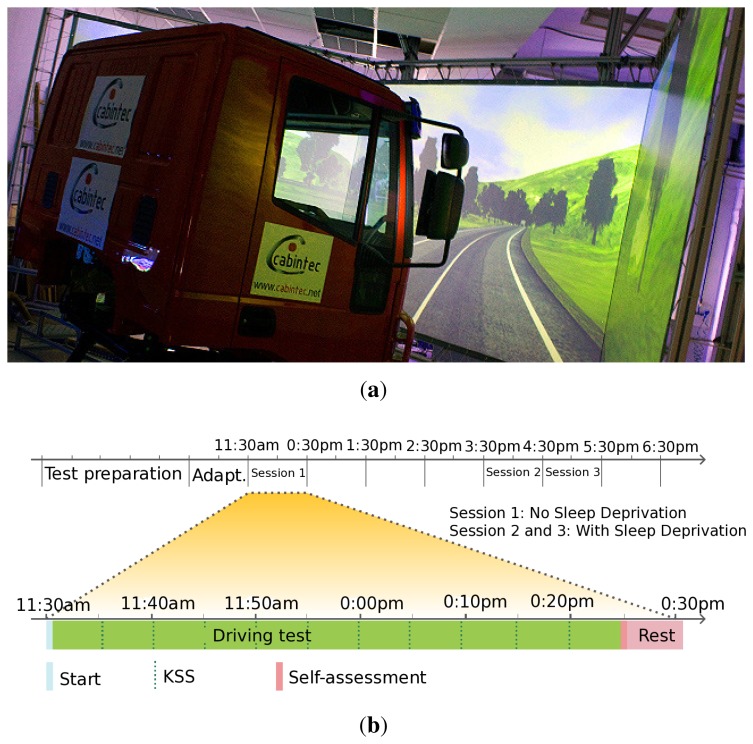
Simulation test design. (**a**) Simulation environment overview; (**b**) Driving sessions schema.

The study was designed such that each driver would carry out some sessions under two different conditions: (1) without sleep deprivation (NSD), after having slept at least seven hours in the night prior to the day of the experiment and starting at 11:30; (2) with sleep deprivation (WSD), after having slept only four hours during the night preceding the experiment and after a normal working day, starting at 15:30 (after having lunch). Each driver carried out one driving session (1 h) for the NSD condition and two driving sessions (2 h) for the WSD condition, as depicted in Figure 1b. There was a small break of a few seconds between sessions 2 and 3 for data recording. Nine drivers participated in the study. A total of 27 driving hours were collected with and without sleep deprivation in a 2/1 proportion. It is worth mentioning that some drowsiness symptoms appeared in six of the test subjects, obtaining very valuable information about driver drowsiness situations.

Simulation methodologies in the field of drivers fatigue research have been proven to be both cost-effective and efficient. Simulation aims at offering to the driver the opportunity to immerse himself in his habitual workplace. In our case, the study employed a third generation simulator called TUTOR and placed at the CEIT (Centro de Estudios e Investigaciones Técnicas de Gipuzkoa) facilities [41]. For a deeper explanation about the simulator, we refer the readers to the authors' publication [42]. A 50 km-long snippet of a sampled actual 12.5 m-wide per direction AP-2 highway, with two 3.75 m-wide lanes, a speed limit of 120 km/h and that was moderately curved was looped during the driving session. All the driving sessions were carried out under a daylight setting, although day and night conditions were very similar in the simulation environment. During the different driving sessions, there was sparse oncoming traffic and some traffic in the same direction in order to encourage monotonous driving and infer driver drowsiness. The simulator registered driving and driver variables. The driving variables are: vehicle-lane lateral position, steering wheel angle, heading error, speed, braking and acceleration, among others. These are signals that measured the motion of the vehicle resulting from the actions of the driver. These signals were registered at 30 Hz. With regard to the driver variables, these were obtained from our own NIR vision system placed in the dashboard, and they showed the driver behavior by his or her face. These driver variables, such as PERCLOS, blink frequency, blink duration, fixed gaze and others, were also registered at 30 Hz.

### 2.2. Ground Truth Generation

To reduce the very high complexity of the drowsiness detection problem, this is usually treated as a binary classification problem in which the data collected during a given driving interval is categorized as an alert or a drowsy driver state. The class assignment is usually based on the driver's own subjective drowsiness estimation or on experts' estimation from the driver and driving indicators analysis. As we pointed out in the introduction, the Karolinska Sleepiness Scale (KSS) [18] is the subjective scale most employed nowadays. The alert class is normally defined as the driving period corresponding to KSS estimates from one to six and the drowsy class to KSS estimates of eight and nine. To obtain a clear separation of the two classes, samples with KSS = 7 are normally discarded. Some authors argue that it is an intrusive method and that both the drowsiness level and the driver cause interference. Then, they say that drivers have difficulty judging their fitness after three hours of monotonous driving [43–45]. Nevertheless, other authors do not consider these arguments and advocate the use of KSS [17,23].

The estimation of drowsiness level by experts has supporters and detractors. On the one hand, this is a non-intrusive method that is easy to implement for a binary classification case. On the other hand, experts must be trained to estimate the levels of drowsiness from the driver and driving indicators, but clues can change among drivers and experts.

Taking into account that there is no consensus in the state-of-the-art respecting the method that generates the ground truth, we propose a supervised KSS method. Basically, it is a fusion of the two existing tendencies in the literature. The binary output of the KSS is fed back by three experts, previously trained in driver drowsiness detection. Each expert classifies each interval as alert or drowsy based on the binary KSS level assigned by the driver, the indicators obtained from the vision-based driver monitoring system and the driving indicators obtained from the vehicle sensors. A voting criterion is employed to assign the final level. To perturb the test subjects as little as possible while collecting a sufficient number of estimations, they were required to provide estimates every 5 min. Hence, each hour of driving provided 12 supervised KSS estimates. A very high correlation between KSS and supervised KSS was obtained in our experiments, having most of the divergences when the drivers were drowsy. In our opinion, the interference of the proposed drowsiness level estimation was very low. However, it must be remarked that the maximum trial duration was three hours, and according to the literature, KSS starts having problems after 3 h.

## 3. Drowsiness Detection Proposal

**Figure 2.**
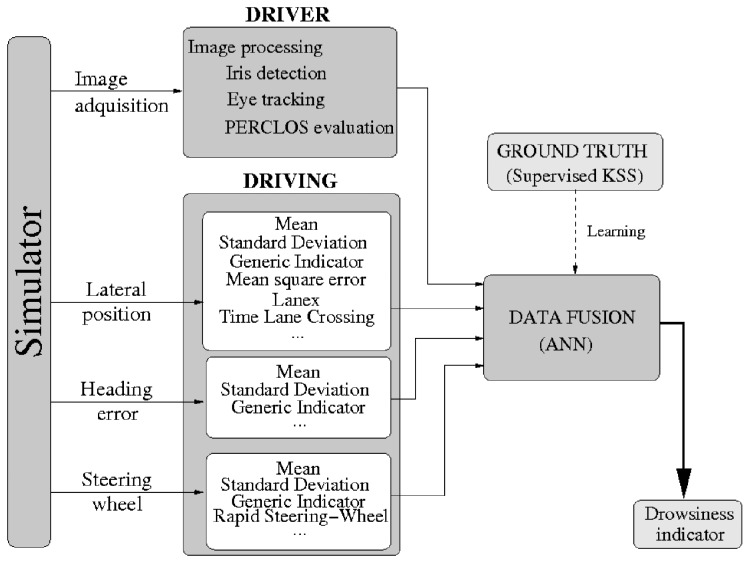
General architecture of our Drowsiness Detection System.

The drowsiness detection is based on ANNs learning and the data fusion of several optimized indicators obtained from driver physical and driving performance signals. Figure 2 shows the general architecture of our proposal.

Driver physical indicators are obtained from our monocular vision system. An NIR illuminator and a camera, sensitive to the NIR, are placed in front of the driver, in the dashboard, in order to acquire images of the driver's face independently of the ambient lighting conditions. When the lighting is low, the NIR illuminator automatically turns on and *vice versa*. Image processing algorithms are used for eye detection and tracking and eye closure parametrization to estimate some driver drowsiness clues.

Driving physical indicators are obtained from the following registered signals in the vehicle: steering wheel movements, heading error and vehicle-lane lateral position. Those signals are normalized, optimized and synchronized in time and space before the computation of the corresponding indicators.

The two kinds of indicators, driver-related and driving-related, are combined using ANNs to get a unique driver drowsiness level. ANNs were chosen, because they infer solutions from data with no prior knowledge of the patterns in the data. Then, this is the fusion technique with the best performance in driver fatigue studies, according to the state-of-the-art. For instance, in some of the previous works [22,44,46,47], this technique retrieves recall rates between 55% and 90%, depending on the inputs. Optimal combinations of a variety of drowsiness indicators proposed in the literature were evaluated by using a conveniently trained ANN. The ground truth was obtained employing the supervised KSS explained in the previous section. Indicators were then subjected to parametric optimization using stochastic optimization methods.

The following paragraphs present the main features of the calculation process for the driver and driving indicators for the proposed system.

## 4. Driver Indicators

Most of the research groups that work on this topic have purchased commercial vision systems for measuring physical driver indicators, such as gaze direction, blink duration, *PERCLOS*, *etc.* Some companies that provide these systems are Seeing Machines, SmartEye or Attention Technology Inc. In this way, researchers can concentrate on exploring the drowsiness detection algorithms instead of the image acquisition and processing. These systems work reasonably well in simulation, but their performance is significantly reduced in real driving conditions.

Due to the fact that the final goal of the authors is to implement our proposal in real vehicles, we have developed our own vision system in order to have full control of this. It provides the following indicators: eye closure duration, blink duration, blink frequency, *PERCLOS*, nodding frequency, face position and fixed gaze. Nevertheless, as the authors showed in [34], the most robust driver physical indicator for real conditions is *PERCLOS*. We experimentally proved that any combination of indicators gave a slight improvement to only using *PERCLOS*. This is the reason why we employed *PERCLOS* as the only driver physical indicator.

*PERCLOS* represents the percentage of time that the eye is more than 80% closed, excluding blinks, per a given period of time, of 30 s in our case, according to our own previous experiments [34]. This indicator is provided by our system in real time (30 Hz). It is user-independent, robust against illumination changes, face gestures or scale and does not require manual calibration. *PERCLOS* measurement for users wearing glasses is not the purpose of this method. Consequently, only drivers without glasses participated in the trials. A deeper explanation of the *PERCLOS* computation methodology proposed by the authors can be found in [48] for simulation and in [49] for real scenarios.

**Figure 3.**
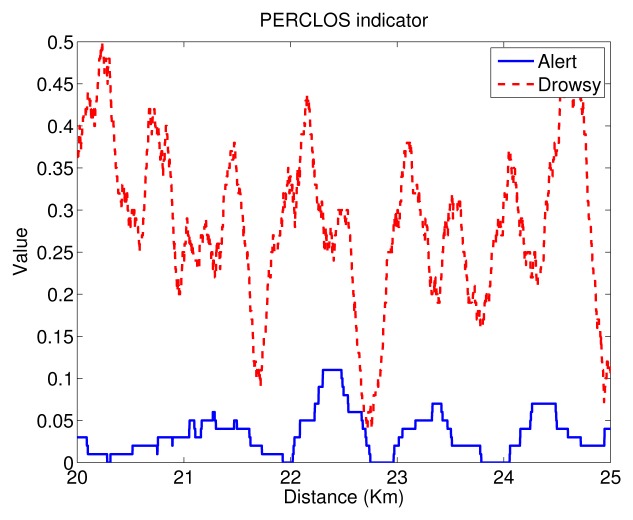
Temporal serie for *PERCLOS*.

For illustration, the evolution of *PERCLOS* is displayed in Figure 3 for a test subject with and without sleep deprivation conditions (drowsy/alert) during a 5-km interval. As can be seen, *PERCLOS* clearly shows higher values for the sleep deprivation condition. In the trial without deprivation, *PERCLOS* varies from zero to 0.11, indicating an alert state. However, in the case of sleep deprivation, *PERCLOS* varies from 0.05 to 0.50, indicating clear drowsiness periods for the highest values.

## 5. Driving Indicators

A variety of drowsiness indicators based on driving behavior has been proposed in the literature. The most important are based on vehicle lateral position in the lane, steering wheel angle and heading error signals [23].

The lateral position signal is measured from a point placed on the right lane boundary to a line (perpendicular to the wheel axes) through the center of the vehicle. The lateral position always takes positive values in the lane. With regard to the steering wheel angle, a value of zero indicates that the steering wheel is centered, and it is positive for the anticlockwise rotation of the wheel. The heading error is defined as the angle between the direction of the heading of the vehicle and the tangent line of the driving lane. A positive error means the vehicle is approaching the left lane boundary and a negative value that it is approaching the right lane boundary.

**Figure 4.**
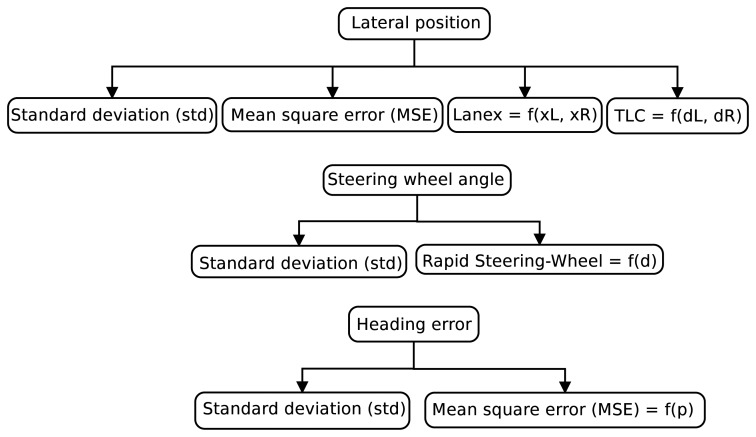
Evaluated indicators.

Figure 4 depicts a scheme of the indicators that are evaluated in this paper. Two studies are carried out:

- Indicators without optimization, with given functional forms and a given parameter setting;
- Optimized indicators, in which the functional form of an indicator from the literature is used, but where the parameters that define the indicator are subject to optimization.

Indicators are calculated by applying windowing techniques over the input signals during 30 s, generating some time series. In this way, the time series scheme employed is the same as the one for *PERCLOS* evaluation.

**Figure 5.**
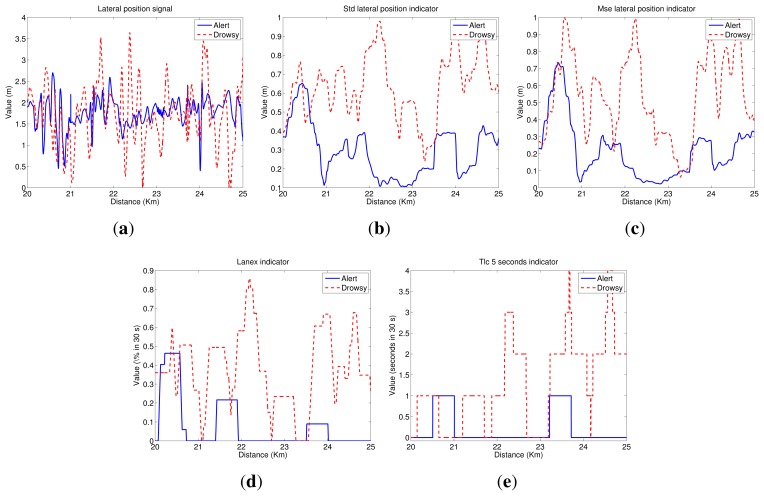
Lateral position indicators. (**a**) Lateral position signal; (**b**) the standard deviation of the vehicle lateral position (*STD_lp*); (**c**) the mean squared error of the lateral position with respect to the center of the driving lane (*MSE_lp*); (**d**) optimized fraction of lane exits (*Lanex_opti*); and (**e**) time to line crossing of 5 s (*TLC_*5 s).

As in the previous section, we show some graphs depicting the driving signal measurements and the indicators associated with them, for a test subject with and without sleep deprivation and driving in the simulator during a 5-km segment. Then, Figure 5 exhibits the lateral position signal and its related indicators. As can be seen, the mean of the lateral position is approximately the center of the lane (1.875 m), and variations are closer to the right lane boundary than the left lane one. Besides, lateral deviation in the drowsy driver is higher than in the alert one, indicating that the drowsy driver has some problems in maintaining the lane direction.

**Figure 6.**
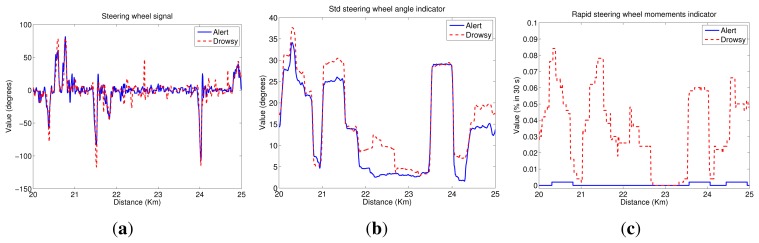
Steering wheel indicators. (**a**) Steering wheel signal; (**b**) the steering-wheel angle (*STD_sw*) and (**c**) rapid steering-wheel movement (*RSWM_opti*).

Figure 6 depicts steering wheel angle signal next to indicators obtained from it. Analyzing the steering wheel angle evolution, we conclude that its values ranged from 90° to −120° for both alert and drowsy subjects, indicating that drivers usually give higher turns to the right lane boundary than to the left lane one. Moreover, if the driver is alert, small corrections are made in the steering wheel, continuously having big variations that correspond to turns due to road curves. If the driver is drowsy, the small variations disappear, and sudden turns are made from time to time to correct the trajectory.

**Figure 7.**
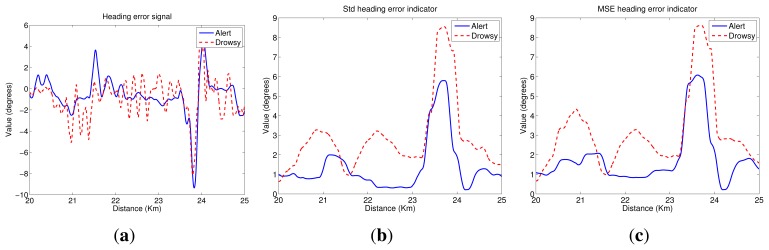
Heading error indicators. (**a**) Heading error signal; (**b**) the standard deviation of the heading error (*STD_he*) and (**c**) the mean squared error of the heading error (*MSE_he*).

Figure 7 depicts the heading error signal and its associated indicators. Heading error values ranged from −10° to 6° for both alert and drowsy drivers. If the driver is alert, these variations correspond to anticipations of road curves, and there is a high correlation with the steering wheel signal. For the drowsy driver, anticipation of the curves is lower, and high variations appear in straight road segments, indicating that the driver has some problems in maintaining the lane direction.

Hereafter, we present the different indicators selected for this study, where *x_i_* represents the i-th sample in a time series and *n* denotes the number of samples used when computing the output value of the indicator. For a deeper explanation of these, we refer the readers to [23].

(1) The standard deviation (STD) of a time series is defined by Equation (1).
  (1) $$\text{STD}=\sqrt{\frac{\sum_{i=1}^{n}{\left(x_{i}-\bar{x}\right)}^{2}}{n-1}}$$
  where *x̅* denotes the arithmetic mean of the time series.
  The standard deviation of the lateral position is, perhaps, the most commonly studied indicator based on driving behavior. However, the standard deviation of other signals, such as the steering-wheel angle and the heading error, has also been studied in the literature [23]. In this paper, we proposed the computation of the standard deviation of the vehicle lateral position (*STD_lp*), the steering-wheel angle (*STD_sw*) and the heading error (*STD_he*).
  Figure 5b plots the *STD_lp* indicator. As can be seen, values for the drowsy test subject are higher than for the alert one. Then, there is a high correlation between these results and the *PERCLOS* ones. For the drowsy driver, there are two segments with clear drowsiness symptoms (21.5–22.5 km and 24–25 km). Figure 6b shows the *STD_sw* indicator. In this case, there is not a high difference between the values for alert and drowsy subjects. Nevertheless, in straight road segments, values for the drowsy driver are slightly higher than for the alert one. Finally, Figure 7b displays the results for *STD_he*, which reaches higher values for the drowsy test subject than for the alert one, even in the case of sharp curves.
(2) The mean squared error (MSE) is defined as the standard deviation in (1), but with the mean value, *x̅*, replaced by a parameter, *p*. For the mean squared error of the lateral position with respect to the center of the driving lane (*MSE_lp*), the expression (*x_i_* − *p*) equals zero when the vehicle is located at the center of the driving lane. Then, the value of *p* is 1.875 m before optimization. The optimization process carried out will be explained in Section 6. The mean squared error of the heading error is denoted as *MSE_he*. When (*x_i_* − *p*) equals zero, this means that the heading of the vehicle and the tangent line of the driving lane are the same. Thus, *p* equals zero.
  Figure 5c shows the *MSE_lp* indicator. As can be appreciated, this indicator is highly correlated with *STD*_*lp*; then, the same conclusions can be drawn. Figure 7c depicts *MSE_he* and a similar behavior as the *STD_he* indicator can be observed.
(3) The fraction of lane exits (*Lanex*) is a measure of a driver's tendency to exit the lane [50]. It is defined as the fraction of a given time interval spent outside the driving lane. The *Lanex* indicator depends on the lateral position signal and is computed from Equation (2).
  (2) $$\text{Lanex}=\frac{\sum_{i=1}^{n}\theta (x_{i})}{n}$$
  where:
  (3) $$\theta (x_{i})=\{\begin{array}{ccc} 1 & if & x_{i}>x_{L}\\ 1 & if & x_{i}<x_{R}\\ 0 & & \text{otherwise} \end{array}$$
  Here, *x_L_* = 2.625 m and *x_R_* = 1.125 m define the real left lane boundary and right lane boundary, respectively, such that *x_i_* = *x_L_* corresponds to a situation in which the left wheels of the vehicle (a truck 2.25 m-wide) touch the left lane line. Similarly, at *x_i_* = *x_R_*, the right wheels touch the right lane line. In a general way, the parameters (*x_L_*, *x_R_*) define the positions of virtual lines placed anywhere on the road. The position of these lines can be optimized in order to improve *Lanex* performance obtaining *Lanex_opti.*
  Figure 5d plots the results for *Lanex_opti* instead of *Lanex*, because the vehicle never crosses the real lane for both test subjects. Once the parameters (*x_L_*, *x_R_*) are optimized, the virtual lines are overtaken for both drivers. In this case, *Lanex_opti* for the drowsy subject has quite higher values than for the alert one. Then, there is a high correlation with the previous indicators, the difference between indicators in this last case for alert/drowsy being higher, due to the integrate values of a binarization process controlled by a threshold.
(4) The time to line crossing (TLC) is defined as the time needed for the vehicle to cross any of the lane boundaries (left or right) [51]. Although *TLC* can be accurately calculated using trigonometry, approximations are commonly employed due to the complexity of these operations and the problem of obtaining the required variables. In this work, the first derivative of the lateral position is applied, *i.e.*, the lateral speed (*ẋ*). When *ẋ* is positive, this corresponds to a lateral movement of the vehicle towards the left boundary of the driving lane, and when it is negative, this corresponds to movements towards the right boundary. The *TLC* is calculated using the following equation:
  (4) $$\text{TLC}=\{\begin{array}{ccc} d_{R}/\dot{x} & if & \dot{x}<0\\ d_{L}/\dot{x} & if & \dot{x}>0 \end{array}$$
  where *d_R_* = 1.125 − *x_i_* is the (negative) distance from the right boundary of the virtual driving lane to the right side of the right front wheel of the vehicle, and similarly, *d_L_* = 2.625 − *xi* is the distance from the left boundary of the virtual driving lane to the left side of the left front wheel of the vehicle. Note that *TLC* is mathematically undefined for *ẋ* = 0. To avoid *TLC* values approaching infinity as *ẋ* approaches zero, a constant *TLC_max_* is introduced, such that *TLC_i_* is set to *min*(*TLC_i_*,*TLC_max_*), where *TLC_i_* denotes sample *i* in the time series. On the other hand, for *d_L_* < 0 and for *d_R_* > 0, *TLC_i_* is set to zero, because it represents that the vehicle is out of the virtual lane.
  Here, we will use an indicator based on [52] and defined as the number of times that the *TLC* signal falls below 5 s in a given time interval (*TLC*_5*s*), which is presented in Equation (5).
  (5) $$\text{TLC}\_5s=\sum_{i=1}^{n-1}f({\text{TLC}}_{i})$$
  where:
  (6) $$f({\text{TLC}}_{i})=\{\begin{array}{ccc} 1 & if & {\text{TLC}}_{i}<a\\ 0 & & \text{otherwise} \end{array}$$
  With *a* = 5. This parameter can be adjusted in an optimization process generating an optimized TLC (*TLC_opti*). Another important TLC-based indicator is the one proposed in [51], which measures the average of TLC (*TLC*_*avg*) according to the following expression:
  (7) $$\text{TLC}\_\text{avg}=-\frac{1}{n}\sum_{i=1}^{n}{\text{TLC}}_{i}$$
  In Figure 5e, the *TLC_*5*s* indicator is depicted. In practice, as in the *Lanex* case, the virtual lane obtained from the optimized parameters is employed. As can be seen, the results are quite correlated to the *Lanex_opti* plot (Figure 5d).
(5) Rapid steering-wheel movement (RSWM) measures the fraction of the steering-wheel velocity that exceeds a specified threshold value during a given time interval. This indicator is calculated by applying the following equation:
  (8) $$\text{RSW}\;M=\frac{1}{n-1}\sum_{i=2}^{n}h({\dot{s}}_{i})$$
  where *ṡ* is the first derivative of the wheel angle signal and:
  (9) $$h({\dot{s}}_{i})=\{\begin{array}{ccc} 1 & if & \left|{\dot{s}}_{i}\right|>d\\ 0 & & \text{otherwise} \end{array}$$

The value of the threshold, *d*, which was proposed in [50], was initially set as 125°/*s*. Subjecting *d* to optimization (*RSWM_opti*), a value of 13°/*s* is obtained.

Figure 6c depicts the *RSWM_opti* indicator results. Values for the drowsy subject are clearly higher than for the alert one. Information derived from the alert driver indicates that the threshold is not usually exceeded. This indicator is highly correlated to that derived from the lateral position signal for the drowsy driver, but it is almost zero for an alert driver. In this way, it is easy to differentiate between the two conditions, although the threshold definition is critical.

## 6. Indicator Optimization

**Figure 8.**
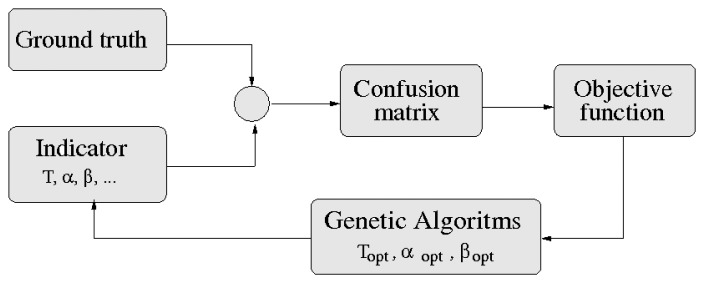
Optimization process.

The indicators analyzed in the previous sections have some constant parameters whose values are set by their proponents in an experimental way. However, there is no guarantee that these parameters are optimized to maximize drowsiness detection. The optimization process finds values for the parameters of a drowsiness indicator, such that the system's ability to distinguish between a drowsy or an alert driver is maximized. The optimization process is depicted in Figure 8.

As mentioned in Section 2, the problem of drowsiness detection has been treated as a binary classification (alert *vs.* drowsy). Besides, a supervised KSS method has been proposed to generate a binary ground truth for the optimization process.

When optimizing a classifier, one must take into account that the size of the dataset is finite and there exists a risk of fitting to noise. For avoiding overfitting, a dataset that is sufficiently large and a holdout validation is employed. In this procedure, the data is divided into three subsets: training, validation and testing. The training subset is employed to optimize the parameters; the validation data helps in determining the quality of the training and the stopping criteria, and finally, the testing subset allows for the evaluation of the actual classifier performance.

As mentioned in Section 2, nine test subjects participated in the study. The ground truth signal was generated every 5 min. Thus, in each hour of a driving session, 12 supervised KSS estimates were obtained. Some intervals had to be removed from the data, either because of simulator/vehicle loss of data or because of driver problems during the session. After removing those intervals, the final number of supervised KSS estimates was 314. Note that the indicators were calculated over a time series of 30 s (*i.e.*, 900 samples). Thus, the time series that correspond to a supervised KSS estimate (300 s) were split into 10 time series of 30 s in length. As a consequence, each supervised KSS estimate provided a total of 10 data points. Then, the entire dataset in simulation contained a total of 3,140 data points, 60% for training, 20% for validation and the remaining 20% for testing.

For binary classification, it is mandatory to apply a threshold, *T*, to the output values of each indicator. Then, the class assignment is obtained applying Equation (10).

(10) $$C=\{\begin{array}{ccc} \text{Drowsy} & if & \text{Indicator}>T\\ \text{Alert} & & \text{otherwise} \end{array}$$

Prior to the application of the objective function, a confusion matrix is computed as depicted in Figure 8. The objective function is defined by Equation (11):

(11) $$f=\frac{\Gamma^{\text{sens}}+\Gamma^{\text{spec}}}{2}=\frac{1}{N}\sum_{i=1}^{N}\frac{\Gamma_{i}^{\text{sens}}+\Gamma_{i}^{\text{spec}}}{2}$$

where Γ*^sens^* is the sensitivity and Γ*^spec^* is the specificity for all the test subjects (*N*). They are obtained from the confusion matrix through the following expressions for each subject, *i*:

(12) $$\begin{array}{cc} \Gamma_{i}^{\text{sens}}=\frac{TP}{TP+FN} & \Gamma_{i}^{\text{spec}}=\frac{TN}{TN+FP} \end{array}$$

where TP (true positive) is the number of data points classified as the drowsy state, FN (false negative) is the number of data points belonging to the drowsy class and classified in the alert one, TN (true negative) is the number of correctly classified data points in the alert class and FP (false positive) is the number of data points belonging to the alert class and classified as drowsy. Note that *f* takes values in the range [0,1]. It is well known that there are individual differences in the effects of drowsiness on driving behavior. In this paper, we try to minimize the effects of individual differences.

A stochastic optimization algorithm is chosen, *i.e.*, a genetic algorithm (GA) [53], for optimizing the indicators. A detailed description of the GA workings is out of the scope of this paper. The values obtained in this optimization process for the parameters of the indicators are shown in Table 2.

## 7. Data Fusion

A drivers behavior is complex, variable and non-linear. Establishing dependences between driver-related and driving-related indicators with the driver drowsiness level is a complex task. Artificial neural networks (ANNs) have been studied and utilized in numerous drowsiness studies. One of the main advantages of ANNs is that they infer solutions from data with no prior knowledge of the patterns in the data. This characteristic is very important, because in driver behavior studies, the exact input-output relationship is difficult to establish. ANNs also have the ability to generalize (*i.e.*, they respond with a reasonable accuracy to patterns that are broadly similar to the original training patterns), which is very useful, because real-world data is noisy, distorted and often incomplete. ANNs are nonlinear, which allows them to solve some complex problems more accurately than linear techniques [54].

**Figure 9.**
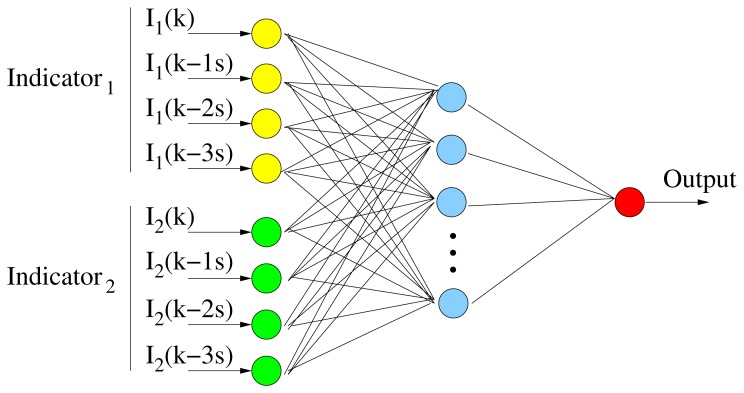
The designed feed forward neural network.

In this work, the feed-forward neural network (FFNN) architecture, one of the most important types of ANNs, is employed, as depicted in Figure 9. FFNNs have a layered structure, where each layer consists of neurons receiving their inputs from neurons of a layer directly below and sending their outputs to units in a layer directly above the unit. There are no connections within a layer. In our case, the input signals were passed through a layer of hidden neurons. Next, the output signals from the hidden neurons were passed through a single output neuron, thus generating the output of the FFNN. A sigmoid transfer function has been used for both hidden and output layers. Thus, all neurons, including the output neuron, provided output values in the range [0,1]. In order to train the FFNN, a scaled conjugate gradient back-propagation method is used.

Different network configurations were analyzed relating the number of the hidden neurons, the time series and the different inputs. As the first approach, indicators were provided to the FFNN at the rate of one every 30 s. In this case, an input neuron was set for each indicator. However, this topology lacked memory, due to only one value being accounted for, for each classification. Therefore, to improve classification performance, some memory was added to the input. The best results were achieved for the topology in Figure 9. For each indicator, four inputs are provided to the FFNN: *I*(*k* − *t*), which is the indicator shifted *t* seconds, where *t* = 0, 1, 2, 3. They are computed in a 30-s interval and shifted 1 s between them. Considering that the average vehicle velocity is about 90 km/h, the data samples of the last 100 m are analyzed in order to generate each FFNN output (drowsy or alert). This topology represents a trade-off between the desire to reduce the sensitivity to small variations, as well as corrective maneuvers lasting a few seconds and the desire to obtain a fast detection performance.

Different combinations of indicators as inputs to the FFNN are analyzed in the experimental results section. With respect to the optimum number of hidden neurons, a ROC (receiver operating characteristic) curve is computed for a number of neurons in the hidden layer from five to 100, adding five each time. The best performance is obtained for 10 neurons. A model for all the subjects is built in order to minimize the effects of individual features. Therefore, the data from all the subjects are merged together, and a model is trained on the whole dataset available. On the other hand, the entire dataset has been divided in the same way as described in the indicator optimization section.

## 8. Experimental Results

This section presents the experimental results obtained in simulation. As stated in previous sections, our goal is to develop a drowsiness model for all test subjects minimizing the effects of individual differences. All the results are presented according to this consideration. It is possible that one might have obtained even better results by developing a model for each individual, *i.e.*, by separately running the optimization and fusion process for each test subject, but it is out of the scope of this paper for two main reasons. The first one is that the vehicle would have had to know the identity of the driver to estimate drowsiness, complicating the detection system. The second and more important objection concerns the amount of data. With only a total of around 300 data points per individual, of which around 180 would be used for training and the rest for validation and testing, the risk of overfitting would significantly increase. In addition, with only about 60 points per individual available for testing, the test results are very sensitive to random noise [23]. Although individual variation has not been specifically addressed during optimization nor fusion, it is possible to measure the performance of a given indicator or combination for each test subject. The figures in this section present several results in order to compare the subjects with the highest drowsiness symptoms over those obtained for all the users. All the results presented in this section have been obtained for the validation dataset.

Considering the ground truth signal, 70.9% of the cases are classified as alert and 29.1% as drowsy for the whole simulation trial. Table 3 depicts the percentage of data points from each test subject, *U_i_*, in the two states.

Indicators described in Sections 3 and 4 are calculated for the whole test subject trials in two conditions: with and without sleep deprivation; so, they are used as inputs of the ANN classifier. In order to evaluate the performance of each proposal (with and without optimization), the sensitivity and specificity are computed for the binary classification into alert or drowsy. The results obtained for all the users taking the parameters (if any) of the indicators specified in the literature, that is, without optimization, are depicted in Table 4. As shown in the table, the performance (over the test set), measured using the objective function, *f* (see Equation (11)), ranges from 0.37 to 0.75 for the indicators based on driving behavior signals, whereas a score of 0.86 is obtained from the *PERCLOS*. The driver behavior indicator, *PERCLOS*, obtains the best results, followed by the driving behavior indicator, *MSE_he*. Table 4 also shows results corresponding to test subject U7; that is, indicators trained over the whole training set, but tested over the U7 test set. In general, the recall rate for each indicator stays in a rather narrow range around the mean for most individuals. However, for U7, the results are slightly better, because his behavior is quite standard and follows the general tendency of most of the test subjects. These have been the reasons why U7 has been chosen to be compared with the mean of all of the subjects. The *f* values range from 0.44 to 0.83 for the indicators based on driving behavior signals, the best being *MSE_he*, whereas a score of 0.9 is obtained from the *PERCLOS* indicator.

Values below 0.5 in the objective function, *f*, represent a random classification, which lacks interest, so only values with the highest scores might be considered. These indicators are: *PERCLOS*, *MSE_he*, *STD_he*, *TLC_avg* and *MSE_lp*.

Applying the optimization techniques explained in Section 6, the new indicators yield the results in Table 5. The performance of these optimized indicators (over the test set) ranges from 0.65 to 0.80 for all the test subjects and from 0.69 to 0.86 for U7. These indicators slightly improve the values in Table 4. As can be seen in Table 5, the best results for all subjects are reached for the *MSE_he_opti* indicator with an *f* value of 0.80, followed by the *Lanex_opti* indicator with 0.75. Again, the values for U7 are higher for the same reasons argued before.

The first indicator in Table 5, (*MSE_lp_opti*), which optimizes the mean squared error for the lateral position, obtained its best performance with *p* = 1.865 m, which is only 10 mm to the right of the actual center of the driving lane, making results for *MSE_lp* very similar to those obtained for *MSE_lp_opti*. For the case of *MSE_he_opti*, the best performance is obtained with *p* = −1.2, which means that there is a small angle towards the right between the heading of the vehicle and the tangent line of the driving lane, indicating that drivers usually drive towards the right boundary of the driving lane. For *Lanex_opti*, the best values achieved are obtained for *x_L_* = 2.27 m and *x_R_* = 1.42 m. They correspond to the left virtual line being placed 0.35 m to the right of the left boundary of the driving lane and the right virtual line being place 0.30 m to the left of the right boundary of the driving lane. Thus, both virtual lines are placed inside the driving lane by the optimization algorithm. The best performance for *TLC_opti* is reached for *a* = 6.4, which is quite higher than the reference parameter (*a* = 5), because the vehicle was a truck. For *RSWM_opti*, the best results are obtained with *d* = 13°/*s*, significantly lower than the reference value (125°/*s*).

Once indicators have been optimized, the next step in the development of the drowsiness system is the fusion of them. All the indicators of Table 5 are combined with the best unoptimized indicators shown in Table 4. As a result, Table 6 depicts the best combination tested for all the subjects, in general, and U7, in particular.

All the indicators show better results when combined with others in pairs. However, combinations between driving-related indicators show only mild improvements. The highest detection performances are obtained combining *PERCLOS* with other driving-related optimized indicators. *PERCLOS* and *MSE_he_opti* or *MSE_lp_opti* combinations yield an *f* value of 0.96 and 0.93, respectively. However, for more than two indicators, these values do not increase. An example is shown in Table 6, where the objective function, *f*, is 0.91, probably due to the excessive number of entries.

As a conclusion, a single driving behavior optimized indicator combined with *PERCLOS* is sufficient to reach the best performance found in this paper, *i.e.*, a score of 0.96 on previously unseen data.

## 9. Conclusions and Further Work

A non-intrusive driver drowsiness system has been proposed, which employs the fusion of several optimized indicators based on driver physical and driving performance measures in simulation.

In the ground truth generation process, a very high correlation between KSS and supervised KSS is obtained in our experiments, most of the divergences occurring when the drivers are drowsy. Then, our proposal validates the KSS method over our experiments and slightly improves the classification when drowsiness symptoms appear in the drivers. In our opinion, the drowsiness interference level of this methodology is very low. However, we have to remark that our maximum trial duration was three hours, and according to the literature, KSS starts having problems after three hours.

Experiments were carried out in an advanced third generation driving simulator. The data was collected from three driving sessions of 1 h for each test subject with varying degrees of drowsiness. The tests presented in this work are oriented toward professional truck drivers, which may present a slightly different behavior compared to non-professional ones, although the system can be adapted to these cases.

Different drowsiness indicators (based on driver and driving behavior) proposed in the literature are evaluated. *PERCLOS* has been proven to be highly correlated to the ground truth, being the best indicator obtained from the driver physical and driving performance measures. *PERCLOS* has been computed from our own vision system, yielding results equal to, or even better than, other commercial systems [37,38] and being more flexible. The recall rate is above 0.86, which is better than other recent works, like [23].

To improve performance, indicators were subjected to parametric optimization using stochastic optimization methods. Hence, the best performing drowsiness indicators were combined between them by using an ANN. The best fusion was *PERCLOS* + *MSE_he_opti*. These results are, in general terms, similar to [23], except the use of *PERCLOS*, but better than other works on the state-of the-art, such as [45] or [55].

Our goal was to develop a drowsiness model for all test subjects, minimizing the effect of individual differences. In general, we found that the recall rate for each indicator stays in a rather narrow range around the mean for most individuals. A separate training of the classifiers for each test subject was not carried out for the overfitting risk, due to the low number of data points per subject.

During the development of this system, some improvements have been identified to be studied in the near future.

Regarding our vision system, it does not work correctly with users wearing glasses, due to light glare and reflections. Consequently, we set the prior requirement for the system evaluation of test subjects to not wearing glasses. Nevertheless, this limitation can be overcome by developing a glasses detector and disabling *PERCLOS* measurements in the fusion for those drivers wearing glasses. Then, only driving indicators would be taken into account, yielding an estimated 10% performance decrease for users wearing glasses. Hence, our proposal could be generalized for every driver.

Even though the technique employed in the fusion process is a feed-forward neural network (FFNN), the problem is open to other neural network topologies and other classification methodologies, using techniques, such as active learning.

A longer database would be necessary to validate our proposal in a deeper way. Having a huge database would allow us to explore the separate training of the classifiers for each test subject and to compare the results with those obtained for all the subjects.

Finally, the authors have the intention of validating the proposed methodology in real conditions. We have already carried out some tests, and the results, as well as a comparison with the simulation results, will be published in the near future.

## Figures and Tables

**Table 1.** Distribution and main features of driving trials. NSD, no sleep deprivation; WSD, with sleep deprivation.

**Trials**	**Simulation** (2.1)
Number of users	9
Conditions	with & without sleep deprivation
No. of sessions	1 (NSD) 2 (WSD)
Session duration	1h1h
Characteristics	Simulator (AP-2 highway)
Brand Vehicle	IVECO (Industrial VEhicle COrporation) Stralis

**Table 2.** Optimized indicators parameters. Lanex, fraction of lane exits; TLC, time to line crossing; RSWM, rapid steering-wheel movement.

	**MSE_lp**	**Lanex**		**TLC**	**RSWM**	**MSE_he**

**Trial**	**p**	**x_L_**	**x_R_**	**a**	**d**	**p**
Simulation	1.865 m	2.27 m	1.42 m	6.4 s	13°/s	−1.2°

**Table 3.** Percentage of samples in each class per driver (simulation).

**Class**	**U1**	**U2**	**U3**	**U4**	**U5**	**U6**	**U7**	**U8**	**U9**
Alert	92	68	55	100	63	97	29	69	65
Drowsy	8	32	45	0	37	3	71	31	35

**Table 4.** Indicators without optimization (simulation).

	**All users**			**User U7**		

**Indicator**	Γ^spec^	Γ^sens^	**f**	$\Gamma_{7}^{\text{spec}}$	$\Gamma_{7}^{\text{sens}}$	**f**
Lateral Position	0.89	0.12	0.50	0.92	0.18	0.55
STD_lp	0.63	0.58	0.61	0.74	0.68	0.71
MSE_lp	0.78	0.50	0.64	0.84	0.59	0.72
Lanex	0.96	0.08	0.51	0.99	0.15	0.57
TLC 5 s.	0.97	0.23	0.60	0.99	0.28	0.63
TLC_avg	0.76	0.62	0.69	0.86	0.71	0.79

Steering Wheel	0.09	0.66	0.37	0.20	0.75	0.47
STD_sw	0.86	0.01	0.43	0.98	0.01	0.49
RSWM	0.79	0.03	0.41	0.84	0.03	0.44

Heading error	0.33	0.47	0.40	0.45	0.56	0.50
STD_he	0.88	0.53	0.70	0.98	0.62	0.80
MSE_he	0.94	0.56	0.75	0.97	0.68	0.83

PERCLOS	0.90	0.82	0.86	0.92	0.89	0.90

**Table 5.** Indicators with optimization (simulation).

	**All users**			**User U7**		

**Indicator**	Γ^spec^	Γ^sens^	**f**	$\Gamma_{7}^{\text{spec}}$	$\Gamma_{7}^{\text{sens}}$	**f**
MSE_lp_opti	0.91	0.47	0.69	0.99	0.48	0.73
Lanex_opti	0.90	0.60	0.75	0.99	0.65	0.82
TLC_opti	0.77	0.58	0.68	0.86	0.59	0.73

MSE_he_opti	0.85	0.74	0.80	0.92	0.81	0.86

RSWM_opti	0.81	0.49	0.65	0.84	0.53	0.69

**Table 6.** Possible combinations of indicators (simulation).

	**All users**			**User U7**		

**Indicator**	Γ^spec^	Γ^sens^	**f**	$\Gamma_{7}^{\text{spec}}$	$\Gamma_{7}^{\text{sens}}$	**f**
PERCLOS + MSE_lp_opti	0.98	0.88	0.93	0.98	0.89	0.94
PERCLOS + Lanex_opti	0.98	0.88	0.93	0.99	0.89	0.94
PERCLOS + TLC_opti	0.98	0.82	0.90	0.99	0.82	0.90
PERCLOS + MSE_he_opti	0.98	0.94	0.96	0.98	0.96	0.97

TLC avg + MSE_lp_opti	0.98	0.54	0.76	0.98	0.55	0.76
TLC avg + Lanex_opti	0.96	0.44	0.70	0.97	0.46	0.71
TLC avg + TLC_opti	0.98	0.57	0.77	0.98	0.60	0.79
TLC avg + MSE_he_opti	0.87	0.76	0.81	0.88	0.78	0.83

PERCLOS + Lanex_opti + MSE_lp_opti	0.94	0.89	0.91	0.94	0.90	0.92

## References

[1] Commission of the European Communities White Paper—European transport policy for 2010: Time to decide, 2001. http://ec.europa.eu/transport/themes/strategies/2001_white_paper_en.htm.

[2] European Commission ommunication on the Future of Transport on 17 June 2009. http://ec.europa.eu/transport/strategies/2009_future_of_transport.

[3] European Commission White Paper: Roadmap to a Single European Transport Area—Towards a competitive and resource efficient transport system, 2011. http://ec.europa.eu/transport/themes/strategies/2011_white_paper_en.htm.

[4] Ranney T.A., Mazzai E., Garrott R., Goodman M.J. (2000). NHTSA Driver Distraction Research: Past, Present, and Future.

[5] Lee J., Regan M., Young K. (2009). Defining Driver Distraction. Driver Distraction: Theory, Effects, and Mitigation.

[6] Brill J.C., Hancock P.A., Gilson R.D. Driver Fatigue: Is Something Missing?.

[7] Croo H.D., Bandmann M., Mackay G.M., Rumar K., Vollenhoven P. (2001). The Role of Driver Fatigue in Commercial Road Transport Crashes.

[8] Klauer S.G., Dingus T.A., Neale V.L., Sudweeks J.D., Ramsey D.J. (2006). The Impact of Driver Inattention on Near-Crash/Crash Risk: An Analysis Using the 100-Car Naturalistic Driving Study Data.

[9] ROSPA (2001). Driver Fatigue and Road Accidents. A Literature Review and Position Paper.

[10] Dong Y., Hu Z., Uchimura K., Murayama N. (2011). Driver Inattention Monitoring System for Intelligent Vehicles: A Review. IEEE Trans. Intell. Transp. Syst..

[11] Berka C., Levendowski D.J., Lumicao M.N., Yau A., Davis G., Zivkovic V.T., Olmstead R.E., Tremoulet P.D., Craven P.L. (2007). EEG correlates of task engagement and mental workload in vigilance, learning, and memory tasks. Aviat. Space Environ. Med..

[12] Oron-Gilad T., Ronen A., Shinar D. (2008). Alertness maintaining tasks (AMTs) while driving. Accid. Anal. Prev..

[13] De Rosario H., Solaz J., Rodrguez N., Bergasa L. (2010). Controlled inducement and measurement of drowsiness in a driving simulator. Intell. Transp. Syst. IET.

[14] Baek H.J., Lee H.B., Kim J.S., Choi J.M., Kim K.K., Park K.S. (2009). Nonintrusive biological signal monitoring in a car to evaluate a driver's stress and health state. Telemed. e-Health.

[15] Bal I., Klonowski W., Magjarevic R., Nagel J.H. (2007). SENSATION—New Nanosensors and Application of Nonlinear Dynamics for Analysis of Biosignals Measured by These Sensors. World Congress on Medical Physics and Biomedical Engineering 2006.

[16] Hoddes E., Zarcone V., Smythe H., Phillips R., Dement W.C. (1973). Quantification of sleepiness: A new approach. Psychophysiology.

[17] Akerstedt T., Gillberg M. (1990). Subjective and objective sleepiness in the active individual. Int. J. Neurosci..

[18] Kaida K., Takahashi M., Åkerstedt T., Nakata A., Otsuka Y., Haratani T., Fukasawa K. (2006). Validation of the Karolinska sleepiness scale against performance and EEG variables. Clin. Neurophysiol..

[19] Ingre M., Akerstedt T., Peters B., Anund A., Kecklund G. (2006). Subjective sleepiness, simulated driving performance and blink duration: Examining individual differences. J. Sleep Res..

[20] Wakita T., Ozawa K., Miyajima C., Igarashi K., Itou K., Takeda K., Itakura F. Driver Identification Using Driving Behavior Signals.

[21] McCall J., Wipf D., Trivedi M., Rao B. (2007). Lane change intent analysis using robust operators and sparse bayesian learning. IEEE Trans. Intell. Transp. Syst..

[22] Eskandarian A., Mortazavi A. Evaluation of a Smart Algorithm for Commercial Vehicle Driver Drowsiness Detection.

[23] Sandberg D., Akerstedt T., Anund A., Kecklund G., Wahde M. (2011). Detecting driver sleepiness using optimized nonlinear combinations of sleepiness indicators. IEEE Trans. Intell. Transp. Syst..

[24] Volvo (2012). Volvo Driver Alert Control. http://www.volvocars.com/us/top/yourvolvo/volvoownersinstructionalvideos/pages/volvo-driveralertcontrol.aspx.

[25] Mercedes-Benz (2012). Attention Assist. Early warning for driver drowsiness. http://www.mbusa.com/mercedes/benz/safety#module-3.

[26] Lexus (2012). LS 600h. http://www.testdriven.co.uk/lexus-ls-600h.

[27] Saab (2012). Saab Driver Attention Warning Sytem. http://www.zercustoms.com/news/Saab-Driver-Attention-Warning-System.html.

[28] Ueno H., Kaneda M., Tsukino M. Development of Drowsiness Detection System.

[29] Dinges D. (1998). PERCLOS: A Valid Psychophysiology Measure of Alertness as Assessed by Psychomotor Vigilance.

[30] Vural E., Cetin M., Ercil A., Littlewort G., Bartlett M., Movellan J. (2007). Drowsy Driver Detection Through Facial Movement Analysis. Human-Computer Interaction.

[31] Highway Safety Group (2012). Driver Fatigue, Lane Management & Warning Systems. http://www.driverfatiguemonitor.com/dfm/dfm.html.

[32] Flores M.J., Armingol J.M., de la Escalera A. (2010). Driver drowsiness warning system using visual information for both diurnal and nocturnal illumination conditions. EURASIP.

[33] Ji Q., Yang X. (2002). Real-time eye, gaze, and face pose tracking for monitoring driver vigilance. Real-Time Imaging.

[34] Bergasa L., Nuevo J., Sotelo M., Barea R., Lopez M. (2006). Real-time system for monitoring driver vigilance. IEEE Trans. Intell. Transp. Syst..

[35] Heinzmann J., Tate D., Scott R. Using Technology to Eliminate Drowsy Driving.

[36] Jimenez P., Bergasa L.M., Nuevo J., Hernandez N., Daza I.G. (2012). Gaze Fixation System for the Evaluation of Driver Distractions Induced by IVIS. IEEE Trans. Intell. Transp. Syst..

[37] Smart Eye AB (2012). Smart Eye Pro. http://www.smarteye.se.

[38] Seeing Machines (2012). FaceLAB and DSS. http://www.seeingmachines.com.

[39] Bowman D.S., Schaudt W.A., Hanowski R.J. (2012). Advances in drowsy driver assistance systems through data fusion. Handb. Intell. Veh..

[40] Hanowski R., Bowman D., Alden A., Wierwille W., Carroll R. (2008). PERCLOS+: Moving Beyond Single-Metric Drowsiness Monitors.

[41] CEIT (2012). Centro de estudios e investigaciones técnicas de Gipuzkoa. http://www.ceit.es.

[42] Daza I., Hernandez N., Bergasa L., Parra I., Yebes J., Gavilan M., Quintero R., Llorca D., Sotelo M. Drowsiness Monitoring Based on Driver and Driving Data Fusion.

[43] Schmidt E.A., Schrauf M., Simon M., Fritzsche M., Buchner A., Kincses W.E. (2009). Drivers' misjudgement of vigilance state during prolonged monotonous daytime driving. Accid. Anal. Prev..

[44] Friedrichs F., Yang B. Drowsiness Monitoring by Steering and Lane Data based Features under Real Driving Conditions.

[45] Friedrichs F., Yang B. Camera-Based Drowsiness Reference for Driver State Classification under Real Driving Conditions.

[46] Boyraz P., Acar M., Kerr D. (2008). Multi-sensor driver drowsiness monitoring. Proc. Inst. Mech. Eng. Part D J. Automob. Eng..

[47] Sandberg D., Wahde M. Particle Swarm Optimization of Feedforward Neural Networks for the Detection of Drowsy Driving.

[48] Garcia I., Bronte S., Bergasa L., Hernandez N., Delgado B., Sevillano M. Vision-Based Drowsiness Detector for a Realistic Driving Simulator.

[49] Garcia I., Bronte S., Bergasa L., Almazan J., Yebes J. Vision-Based Drowsiness Detector for Real Driving Conditions.

[50] Wierwille W., Lewin M., Fairbanks R. (1996). Research on Vehicle-Based Driver Status/Performance Monitoring: Part III.

[51] Godthelp H., Milgram P., Blaauw G.J. (1984). The development of a time-related measure to describe driving strategy. Hum. Factors J. Hum. Factors Ergon. Soc..

[52] Fairclough S.H., Graham R. (1999). Impairment of driving performance caused by sleep deprivation or alcohol: A comparative study. Hum. Factors: J. Hum. Factors Ergon. Soc..

[53] Holland J.H. (1992). Adaptation in Natural and Artificial Systems: An Introductory Analysis with Applications to Biology, Control and Artificial Intelligence.

[54] Dong Y., Hu Z., Uchimura K., Murayama N. (2011). Driver inattention monitoring system for intelligent vehicles: A review. IEEE Trans. Intell. Transp. Syst..

[55] Caterpillar (2008). Operator Fatigue Detection Technology Review.

